# Comment on ‘Ablation of LAT2 Transporter Causes Intramuscular Glutamine Accumulation and Inhibition of Fasting‐Induced Proteolysis’ by Espino‐Guarch et al.

**DOI:** 10.1002/jcsm.70037

**Published:** 2025-07-30

**Authors:** Jinglin Li, Jiachen Peng

**Affiliations:** ^1^ Affiliated Hospital of Zunyi Medical University Zunyi China; ^2^ Zunyi Medical University Zunyi China

To the Editor,

We have read with great interest the recent article by Espino‐Guarch et al., ‘Ablation of LAT2 Transporter Causes Intramuscular Glutamine Accumulation and Inhibition of Fasting‐Induced Proteolysis’, a landmark study that significantly advances our understanding of skeletal muscle proteostasis [1]. The authors present a compelling, albeit paradoxical, series of findings. They demonstrate that ablating the LAT2 transporter leads to intramuscular glutamine (Gln) accumulation, which in turn inhibits fasting‐induced proteolysis in an mTORC1‐dependent manner. This apparent preservation of muscle mass, however, comes at a steep and unexpected cost: the protective effect vanishes in complex disease states, and most strikingly, the mice exhibit a premature aging phenotype. This work provides a crucial insight into the intricate balance of metabolic regulation, and its paradoxical nature warrants a deeper discussion of the underlying mechanisms.

One of the most profound paradoxes presented is the dissociated nature of mTORC1 signalling. The authors elegantly show that mTORC1 is recruited to the lysosome, a canonical step for its activation by amino acids, which correctly correlates with the suppression of autophagy and proteasomal degradation [1]. Yet this localized activation fails to produce a corresponding anabolic output. Global protein synthesis is not increased, and phosphorylation of the key translational repressor, 4E‐BP1, is paradoxically decreased [1]—the inverse of what canonical mTORC1 signalling would predict.

We contend that this phenomenon represents a ‘signal‐outcome dissociation’, where the spatial and temporal context of the mTORC1 signal dictates its functional capacity. The chronic, Gln‐driven accumulation of mTORC1 at the lysosome likely constitutes a low‐grade, compartmentalized signal. This localized activity is sufficient to phosphorylate adjacent, catabolism‐related substrates like the ULK1 complex, thereby inhibiting autophagy. However, it is critically insufficient to drive a global anabolic programme. Full anabolic activation, including robust phosphorylation of cytosolic targets like 4E‐BP1 and S6K, requires the synergistic integration of multiple inputs, most notably potent growth factor signalling through the Akt‐TSC‐Rheb axis [2, 3]. In the fasted state, where insulin signalling is minimal, the Gln‐driven signal exists in a functional vacuum. This creates a metabolic stalemate: catabolism is switched off, but anabolism cannot be fully switched on. This highlights a critical principle: the mere activation of a kinase at one cellular location does not guarantee the execution of all its downstream programmes.

The internal paradoxes of the LAT2KO muscle manifest as profound external consequences. The authors frame the concurrently accelerated lipolysis in their mice as a potential study limitation [1]. We posit this is not a limitation but a cornerstone finding that supports a ‘selfish muscle’ hypothesis. By hoarding amino acids and preserving its own protein mass during fasting, the LAT2KO muscle forces a dramatic compensatory response from other tissues. The stark reduction in white adipose tissue (WAT) mass, coupled with elevated plasma free fatty acids and ketone bodies [1], strongly suggests that fat stores are being aggressively catabolized to supply the energy that the rest of the body is not receiving from muscle‐derived amino acids.

This metabolic shift implies the existence of a robust, previously unappreciated muscle‐to‐fat communication axis. We hypothesize that the altered metabolic state of the LAT2KO muscle—characterized by both amino acid imbalance and mitochondrial dysfunction—remodels its secretome, causing the release of myokines that actively drive lipolysis in WAT. Prime candidates for mediating this signal include Fibroblast Growth Factor 21 (FGF21), a well‐established ‘starvation hormone’ secreted in response to metabolic stress [4], and Interleukin‐6 (IL‐6), a myokine known to be released under stress and to promote fat breakdown [5]. Thus, the accelerated lipolysis is likely not a parallel event but a direct consequence of a signalling cascade initiated by the metabolically ‘selfish’ LAT2KO muscle, revealing a new layer of inter‐organ crosstalk.

The failure of LAT2 ablation to protect against muscle wasting in models of cancer cachexia and type 1 diabetes (T1D) is a critical result that underscores the fragility of this protective mechanism [1]. In both cases, we propose that the delicate Gln‐mTORC1 axis is simply overridden by dominant pathological signals.

In cancer cachexia, a state of chronic systemic inflammation, an ‘inflammatory override’ is the likely culprit. Pro‐inflammatory cytokines like TNF‐α and IL‐6 are potent drivers of muscle wasting, activating distinct signalling pathways—primarily NF‐κB and STAT3—that directly upregulate key muscle‐specific E3 ubiquitin ligases, MuRF1 and Atrogin‐1 [6, 7]. These powerful inflammatory signals create an independent and overwhelming drive for protein degradation that the nuanced anti‐catabolic signal from Gln‐mTORC1 cannot withstand.

In the T1D model, the mechanism is an ‘insulin deficiency override’. The absolute lack of insulin, the body's master anabolic hormone, disrupts the system on multiple fronts. First, insulin signalling is required for the proper function and trafficking of a host of nutrient transporters, destabilizing the entire cellular amino acid sensing network [8]. Second, as discussed, insulin provides the critical synergistic signal required for full mTORC1‐mediated anabolism [2, 3]. Without it, the Gln‐driven signal is functionally impotent against the rampant systemic catabolism driven by the hormonal milieu of uncontrolled T1D.

Perhaps the most profound finding is that young LAT2KO mice exhibit a premature aging phenotype, with muscle performance metrics resembling those of much older wild‐type animals [1]. This suggests a ‘Faustian Bargain’: the muscle trades long‐term health and function for short‐term mass preservation.

We propose that this accelerated aging is a direct consequence of the chronic inhibition of cellular quality control. The persistent Gln‐driven mTORC1 activation is a potent suppressor of autophagy and specifically mitophagy—the selective clearance of damaged mitochondria [9]. This hypothesis perfectly explains the authors' mitochondrial data: LAT2KO muscle exhibits significantly impaired respiration, yet mitochondrial DNA content and gross morphology are unchanged [1]. This is the classic signature of an accumulation of dysfunctional, but uncleared, mitochondria [10]. This build‐up of defective, ROS‐producing organelles is a known driver of sarcopenia, leading to reduced ATP production (explaining poor treadmill performance) and triggering a cellular stress response, including the upregulation of the atrophy‐ and age‐associated marker Murf1. The core issue revealed by the LAT2KO model is not the *quantity* of muscle protein, but the crippling failure of its *quality control* machinery.

To dissect these complex phenomena, a multi‐faceted research programme is warranted. First, to test the ‘selfish muscle’ hypothesis, the secretome of LAT2KO myotubes could be analysed via mass spectrometry to identify secreted factors like FGF21. Conditioned media from these cells could then be applied to adipocytes to directly measure effects on lipolysis. Second, to probe the ‘inflammatory override’, in vitro studies treating LAT2KO myotubes with TNF‐α could determine the direct impact on LAT2/4F2hc expression and localization, while ChIP‐seq could probe for NF‐kB/STAT3 binding to the relevant gene promoters. Finally, and perhaps most crucially, to test the ‘Faustian Bargain’, the LAT2KO mouse could be crossed with a mitophagy reporter mouse (e.g., mt‐Keima) to directly visualize and quantify the suppression of mitochondrial clearance in vivo. This would pave the way for preclinical tests to see if this bargain can be reversed by treating young LAT2KO mice with low‐dose, intermittent rapamycin or other autophagy inducers like spermidine [11, 12] to rescue muscle function and prevent premature aging.

In conclusion, the work by Espino‐Guarch et al. is a landmark study that serves as a crucial cautionary tale for the field. It masterfully demonstrates that merely preserving muscle mass by blocking catabolism is not a viable long‐term therapeutic strategy if it comes at the cost of cellular quality control and function. The health of a muscle, and perhaps any tissue, is defined not by its static mass, but by its dynamic homeostatic *flux*. This principle should guide future strategies for muscle wasting diseases, shifting the focus from simple preservation to the ambitious but necessary goal of restoring metabolic balance and resilience.
